# Supplementary material for: A yeast phenomic model for the influence of Warburg metabolism on genetic buffering of doxorubicin
Source: Cancer Metab. 2019 Oct 23;7:9. doi: 10.1186/s40170-019-0201-3 (PMC6806529; doi:10.1186/s40170-019-0201-3)
Supplement: Supplementary file 9 — Additional file 9. GO term-specific heatmaps for REMc/GTF-enriched clusters. GO term-specific heatmaps for significant GO process terms were generated as described in methods and Figs. 3 and 4. Any related child terms are presented in subsequent pages of the parent file name. GO terms with more than 100 children, with 2 or fewer genes annotated to the term, or a file size over 300KB are not shown. All heatmaps are generated with the same layout (see Figs. 3 and 4). [file 40170_2019_201_MOESM9_ESM.bz2 › Additional_File9_GOTermHeatmaps/Additional_File9_GOTermHeatmap/ribosome_assembly.pdf]

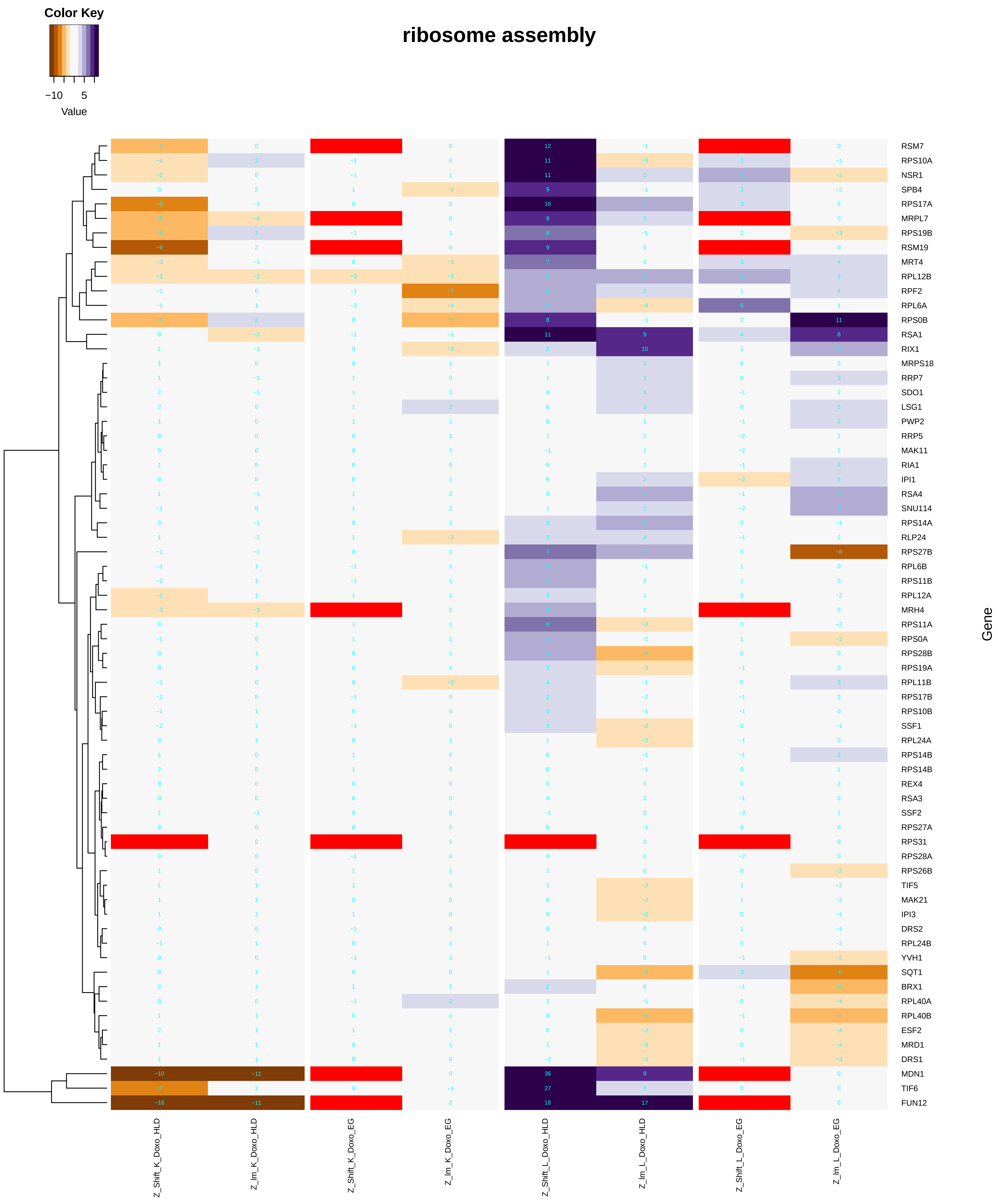

ribosome assembly
Color Key
−10
5
Value
RSM7
RPS10A
NSR1
SPB4
RPS17A
MRPL7
RPS19B
RSM19
MRT4
RPL12B
RPF2
RPL6A
RPS0B
RSA1
RIX1
MRPS18
RRP7
SDO1
LSG1
PWP2
RRP5
MAK11
RIA1
IPI1
RSA4
SNU114
RPS14A
RLP24
RPS27B
RPL6B
RPS11B
RPL12A
MRH4
RPS11A
RPS0A
RPS28B
RPS19A
RPL11B
RPS17B
RPS10B
SSF1
RPL24A
RPS14B
RPS14B
REX4
RSA3
SSF2
RPS27A
RPS31
RPS28A
RPS26B
TIF5
MAK21
IPI3
DRS2
RPL24B
YVH1
SQT1
BRX1
RPL40A
RPL40B
ESF2
MRD1
DRS1
MDN1
TIF6
FUN12
Gene
Z_Shift_K_Doxo_HLD
Z_lm_K_Doxo_HLD
Z_Shift_K_Doxo_EG
Z_lm_K_Doxo_EG
Z_Shift_L_Doxo_HLD
Z_lm_L_Doxo_HLD
Z_Shift_L_Doxo_EG
Z_lm_L_Doxo_EG

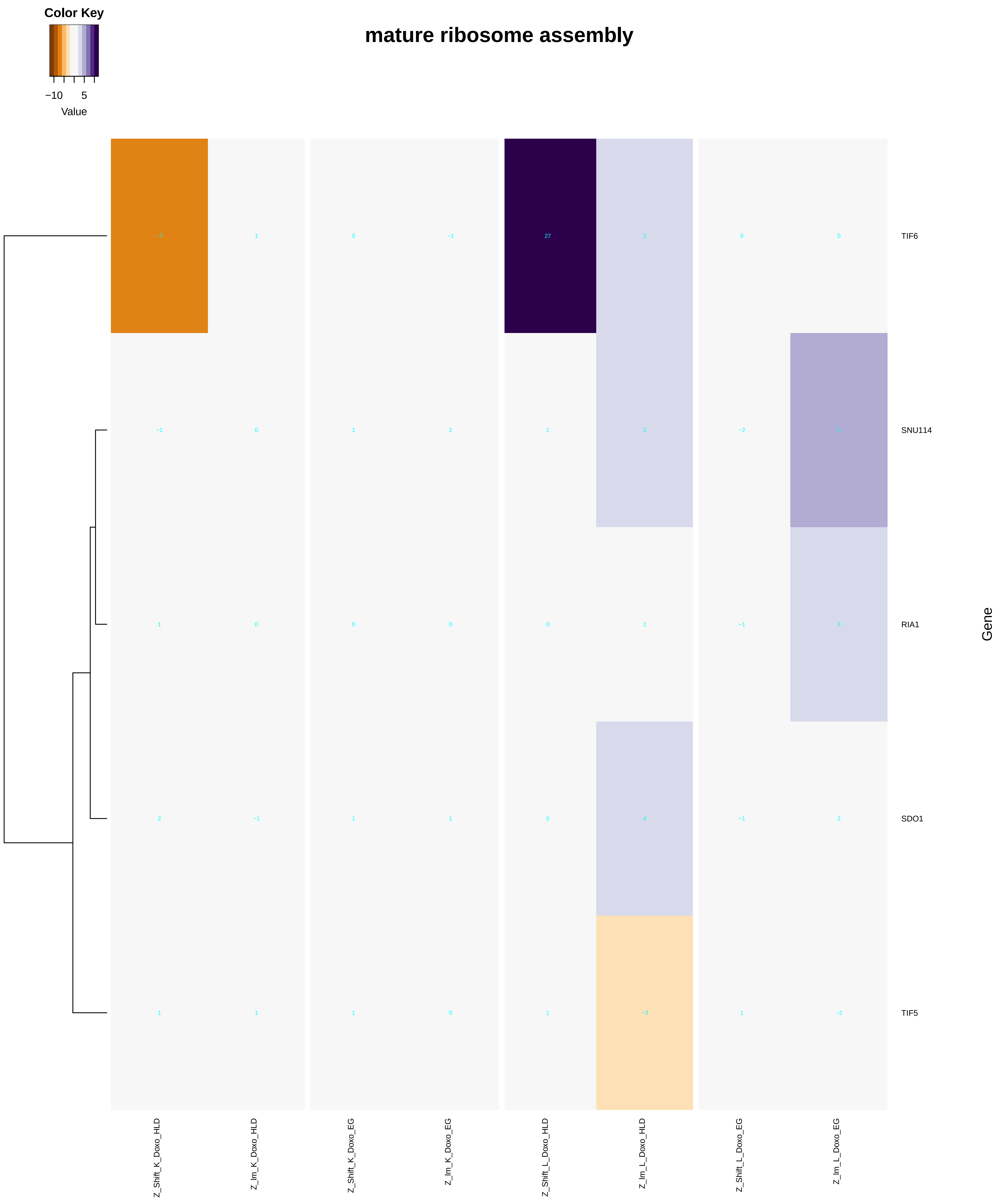
Color Key
−10
5
Value
mature ribosome assembly
TIF6
SNU114
RIA1
SDO1
TIF5
Gene
Z_Shift_K_Doxo_HLD
Z_Im_K_Doxo_HLD
Z_Shift_K_Doxo_EG
Z_Im_K_Doxo_EG
Z_Shift_L_Doxo_HLD
Z_Im_L_Doxo_HLD
Z_Shift_L_Doxo_EG
Z_Im_L_Doxo_EG
